# Preparation and Properties of Branched Polystyrene through Radical Suspension Polymerization

**DOI:** 10.3390/polym9010014

**Published:** 2017-01-06

**Authors:** Wenyan Huang, Weikai Gu, Hongjun Yang, Xiaoqiang Xue, Bibiao Jiang, Dongliang Zhang, Jianbo Fang, Jianhai Chen, Yang Yang, Jinlong Guo

**Affiliations:** School of Materials Science and Engineering, Changzhou University, Changzhou 213164, China; ellahwy@cczu.edu.cn (W.H.); 15189722821@163.com (W.G.); hjyang0519@cczu.edu.cn (H.Y.); xxq969@cczu.edu.cn (X.X.); zhangdongliang@cczu.edu.cn (D.Z.); fjbiloveyou@126.com (J.F.); jhchen@cczu.edu.cn (J.C.); yangyang@cczu.edu.cn (Y.Y.); 15161162203@163.com (J.G.)

**Keywords:** solvent-free, radical polymerization, suspension polymerization, branched polymer

## Abstract

Radical solvent-free suspension polymerization of styrene with 3-mercapto hexyl-methacrylate (MHM) as the branching monomer has been carried out using 2,2′-azobisisobutyronitrile (AIBN) as the initiator to prepare branched polymer beads of high purity. The molecular weight and branching structure of the polymers have been characterized by triple detection size exclusion chromatography (TD-SEC), proton nuclear magnetic resonance spectroscopy (^1^H-NMR), and Fourier transform infrared spectroscopy (FTIR). The glass transition temperature and rheological properties have been measured by using differential scanning calorimetry (DSC) and rotational rheometry. At mole ratios of MHM to AIBN less than 1.0, gelation was successfully avoided and branched polystyrene beads were prepared in the absence of any solvent. Branched polystyrene has a relatively higher molecular weight and narrower polydispersity (*M*_w.MALLS_ = 1,036,000 g·mol^−1^, *M*_w_/*M*_n_ = 7.76) than those obtained in solution polymerization. Compared with their linear analogues, lower glass transition temperature and decreased chain entanglement were observed in the presently obtained branched polystyrene because of the effects of branching.

## 1. Introduction

Branched polymers have some unique properties, such as lower solution and melt viscosities, increased solubility, and many more terminal groups. It is expected that these branched polymers will be used as polymer rheological modifiers [1,2,3,4]. It was not until 1995 that Fréchet et al. reported the preparation of branched vinyl polymer through self-condensing vinyl polymerization of an inimer (SCVP) [5]. Moreover, star polymers were prepared in one-pot by atom transfer radical polymerization (ATRP) of maleimide inimer and styrene [6]. The SCVP of inimer strategy was also used to prepare branched vinyl polymers through living radical polymerization [7,8,9,10,11,12,13]. To eliminate the tedious preparation process of the inimer, Baskaran and Sherrington’s group prepared branched polymers using commercially available divinyl monomer as the branching agent through anionic polymerization and ATRP, respectively [14,15]. To date, a series of branched vinyl polymers were prepared using divinyl monomer as the branching agent [16,17,18,19,20,21], and star polystyrene was prepared by ATRP of bismaleimide and an excess of styrene [22]. Several groups studied the development of branching [23,24,25,26], and concluded that the limited molecular weight and broad polydispersity of the obtained branched polymers resulted from primary chain residue and intramolecular cyclization [25,26,27]. Moreover, the primary chain residue contained no branching agent unit [28]. It is well known that radical polymerization can be carried out under much milder reaction conditions, so the preparation of branched polymers through radical polymerization is much more attractive and valuable. To date, a series of research works have reported on the preparation of branched polymers through radical polymerization, involving strategies of copolymerization of a vinyl monomer polymerized with a divinyl monomer in the presence of a radical transfer agent to avoid gelation [29,30,31,32,33,34], and polymerization in the presence of a polymerizable chain transfer or chain transfer monomer (CTM) [35,36]. With regard to the copolymerization of vinyl and divinyl monomer, the prepared branched polymers show limited molecular weights and much broader polydispersities [32,34]. Additionally, this polymerization must be performed in diluted media, or gelation will occur (except in aqueous or suspension reaction). In our experience, 4-vinyl benzyl thiol—the reported CTM—exhibited poor storage stability [36]. We designed a methacrylic CTM—3-mercapto hexyl methacrylate (MHM)—and prepared branched polymers through radical polymerization [37,38]. In order to inhibit gelation, organic solvent should also be introduced. A branched polymer with high molecular weight and relatively narrow polydispersity can be prepared through emulsion polymerization in the presence of MHM [39]. It is considered that the particular chain termination mechanism in emulsion reaction plays the determinant role in inhibiting gelation. Additionally, water serves well as a heat exchanging agent. However, the polymers obtained after coagulation of the latex are contaminated by impurities such as the emulsifier and coagulating agent residues, since the emulsifier concentration used in emulsion polymerization is typically as high as 1%–5% in weight of the aqueous phase. Polymerization in aqueous suspension offers many advantages that have led to its wide use in commercial production. Suspension polymerization gives polymers of better purity which are isolated easily; i.e., directly by centrifuging or filtering compared with emulsion polymerization, since the levels of suspension stabilizer are typically less than 1.0% in weight of the aqueous phase, which is much lower than the emulsifier concentrations in emulsion polymerization. Suspension polymerization also offers some unique advantages compared to bulk and solution polymerization, since polymer beads of high purity can be obtained directly and water provides an ideal heat-transfer medium during the reaction.

Since suspension polymerization has so many marked advantages, it is postulated here that beads of branched vinyl polymers with high purity can be prepared directly through radical solvent-free suspension polymerization and simple filtration, and this is the objective of our research here. In addition, we also investigated the effects of branching on glass transition temperature and the rheological properties of branched vinyl polymers.

## 2. Experimental Section

### 2.1. Materials

Styrene (St, analytical grade, Shanghai Chemical Co., Shanghai, China) was distilled under reduced pressure. 2,2′-Azobisisobutyronitrile (AIBN, analytical grade, Shanghai Chemical Co.) was recrystallized in ethanol. Methacrylic acid and *iso*-butyric acid (analytical grade, Shanghai Chemical Co.) were used as received. 3-Mercapto-hexanol (MHA, analytical grade, Shijiazhuang Dali Chemical Co., Shijiazhuang, China) was used without further purification. Poly(vinyl alcohol) with alcoholysis degree of 88% and polymerization degree of 1700 (PVA-1788, Shanghai Chemical Co.) was used as received. Other reagents and solvents made from Shanghai Chemical Co. were directly used as received. MHM and 3-mercapto hexyl-isobutyrate (MHIB) were synthesized according to the literature and obtained purities of 99.3% and 98.5% (HPLC), respectively [37,39]. The ^1^H-NMR spectra of MHM and MHIB with all of the resonances assigned are shown in the Supporting Information (Figures S1 and S2).

### 2.2. Suspension Polymerization

Preparation of branched polystyrene: the aqueous phase containing PVA-1788 (0.75% in weight to water) and the organic phase containing styrene, MHM, and AIBN were mixed in a 100 mL round-bottom flask equipped with a stir bar, and degassed in freeze–pump–thaw cycles five times. The weight ratio of the aqueous phase to the organic phase was 3:1. Polymerization was allowed to proceed at 80 °C for 8 h under an argon atmosphere. The polymer was collected by filtration using a Buchner funnel and dried under vacuum to a constant mass. For preparation of linear analogue, the polymerization procedure was similar to that of branched polystyrene with or without MHIB as the chain transfer agent. These polymerizations and the related polymers are designated using the form St_x_-MHM(MHIB)_y_-AIBN_z_, where the subscripts x, y, and z denote the reagent dosages in mmol.

### 2.3. Fourier Transform Infrared Spectroscopy

FT-IR spectra were recorded on a Thermo Nicolet 6700 spectrometer (Thermo Fisher Scientific, Waltham, MA, USA) with NaCl plates.

### 2.4. Proton Nuclear Magnetic Resonance Spectroscopy

^1^H-NMR spectra were recorded on a Bruker ARX-500 type NMR spectrometer (Bruker, Karlsruhe, Germany) at 25 °C with CDCl_3_ as the solvent and tetramethylsilane as the internal standard.

### 2.5. Triple Detection Size Exclusion Chromatography (TD-SEC)

The molecular weight, polydispersity(PDI), root-mean-square (rms) radius of gyration, and intrinsic viscosity were obtained by TD-SEC detection at 25 °C. The instrumentation consisted of the following: a Waters 1515 isocratic HPLC pump with 5 μm Waters styragel columns (Guard, 0.5 HR, 1 HR, 3 HR, 4 HR, and 5 HR columns, with molecular weight ranges of 100–5000 g·mol^−1^, 500–30,000 g·mol^−1^, 5000–500000 g·mol^−1^, and 50,000–4,000,000 g·mol^−1^), a Waters 717 PLUS auto-sampler, a Waters 2414 differential refractive index (DRI) detector with a wavelength of 880 nm, an 18-angle laser light scattering (MALLS) detector (Wyatt mini-DAWN HELEOS-II, Wyatt, Cal., Goleta, CA, USA) (from 22.4° to 151.4°) with a wavelength of 690 nm and power of 220 W, a Wyatt Visco Star viscometer detector, and a Waters Empower data manager. The eluent was HPLC-grade tetrahydrofuran (THF) delivered at 1.0 mL·min^−1^.

### 2.6. Differential Scanning Calorimetry (DSC)

Sample preparation: polymer sheets of 85 mm × 85 mm × 0.3 mm were formed at 200 °C under pressure. Rectangular films of 25 mm × 5 mm were tailored from the formed sheets. Glass transition temperature (*T*_g_) was measured using differential scanning calorimetry (PerkinElmer DSC 6000, Waltham, MA, USA) under nitrogen atmosphere. The temperature range was ambient temperature to 200 °C. The heating rate of 10 °C·min^−1^, kept for 3 min to eliminate the thermal history, and then cooled down to ambient temperature at a rate of 10 °C·min^−1^. Under the same conditions, a second heating run was applied in order to determine *T*_g_, taken at the inflection point of heat capacity change.

### 2.7. Rheological Measurements

Rheological properties were measured by means of an MCR 301 rheometer (Anton Paar, Graz, Austria) using parallel plate oscillation mode in air atmosphere (25 mm diameter, 2 mm gap). Dynamic frequency sweeps from 0.1 to 100 rad/s were performed under a strain of 1% at 190 °C.

## 3. Results and Discussion

In our previous reports, branched polymers were successfully prepared through radical polymerization in the presence of the chain transfer monomer MHM [37,38]. However, it is essential to introduce enough solvent to dilute the reaction system for soluble branched polymers, or gelation will occur. We hypothesize that it is difficult for heat transfer to occur in bulk polymerization and therefore should be an induction for gelation. Suspension polymerization is actually bulk polymerization in monomer droplet, so the heat transfer should be much more convenient, and branched polymers with high purity are expected to be prepared directly through solvent-free suspension polymerization.

### 3.1. Preparation of Branched Polymers

**Figure 1:**
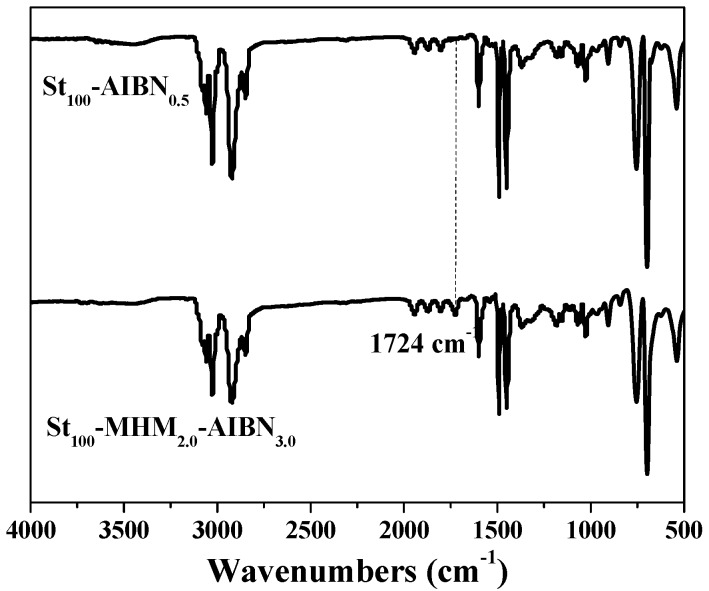
Typical Fourier transform infrared (FT-IR) spectra of the polystyrenes.

**Figure 2:**
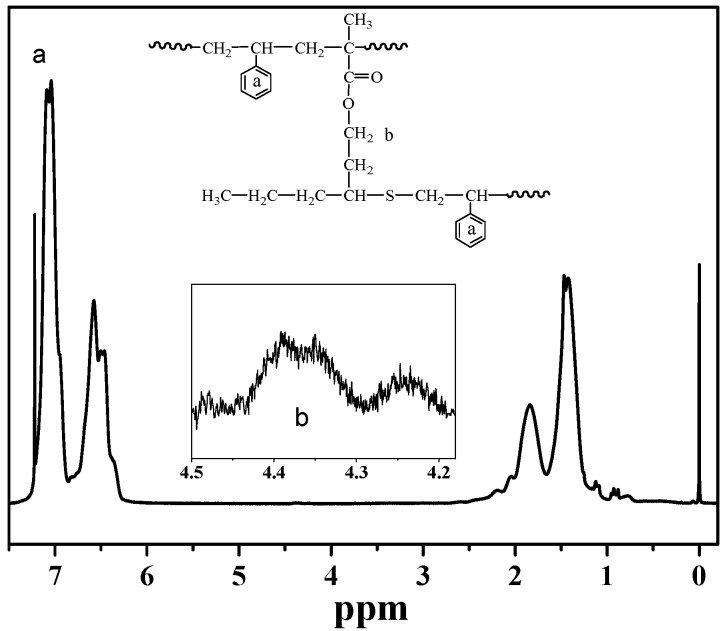
^1^H-NMR spectrum of the polystyrene St_100_-MHM_2.0_-AIBN_3.0_. AIBN: 2,2′-Azobisisobutyronitrile; MHM: 3-mercapto hexyl methacrylate; St: Styrene.

Figure 1 and Figure 2 show the typical FTIR and ^1^H-NMR spectra of the polystyrene prepared through suspension polymerization with and without the chain transfer monomer MHM. The signal at 1724 cm^−1^ in Figure 1 (relating to the carbonyl group, >C=O in MHM) and the signal at around 4.2 to 4.5 ppm in Figure 2 (see inset) (relating to –COO–*CH_2_*– in MHM) prove the incorporation of the MHM unit into the polystyrene; these results are the same as those observed in solution and emulsion polymerization [37,39]. Figure S3 shows the ^1^H-NMR spectrum of the linear polystyrene prepared without MHM and MHIB, but no related signals were observed from 4.0 to 4.5 ppm.

Table 1 illustrates the TD-SEC results of polystyrene prepared using MHM as the branching monomer. When the mole ratio of MHM to the initiator AIBN was less than 1.0, soluble polystyrene could be prepared without gelation. However, when the above mole ratio was greater than 1—as in the cases of branched polystyrene (BPS)-3 and BPS-4—gelation occurred. For selected styrene and MHM concentrations, polymers prepared at higher initiator concentration exhibited lower molecular weights (BPS-5 vs. BPS-8; BPS-9 vs. BPS-10, Table 1). These results should be accounted for by the shorter primary chain length at high initiator concentration. For selected styrene and initiator concentrations, polymers prepared at higher MHM concentration showed higher molecular weights (compare BPS-7 vs. BPS-9 and BPS-10 vs. BPS-11), which are similar results to those that were observed in solution and emulsion reaction because of the availability of some more MHM units per primary chain [37,39].

**Figure 3:**
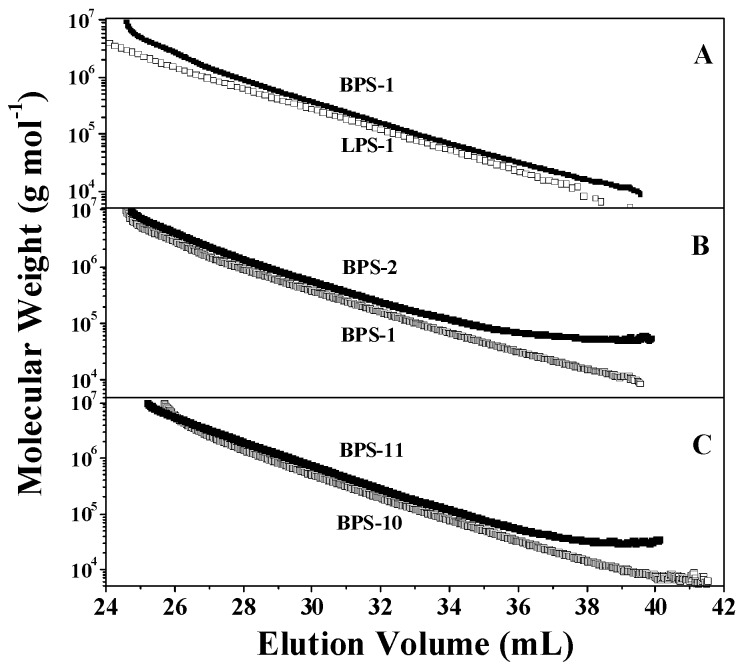
Plots of molecular weight versus elution volume.

Figure 3 illustrates the dependence of molecular weight on elution volume for the polymers prepared with or without MHM. BPS-1 consistently had a larger *M*_W.SEC_ than that of LPS-1 at any selected elution volume, regardless of its very low MHM concentration (Figure 3A), confirming the branching structures of BPS-1 [1,2,3,4,5,40]. For a fixed initiator concentration, polymers prepared at higher MHM concentration exhibited higher molecular weight at given elution volume (Figure 3B,C), indicating that polymers obtained at higher MHM concentration showed some higher degree of branching.

**Figure 4:**
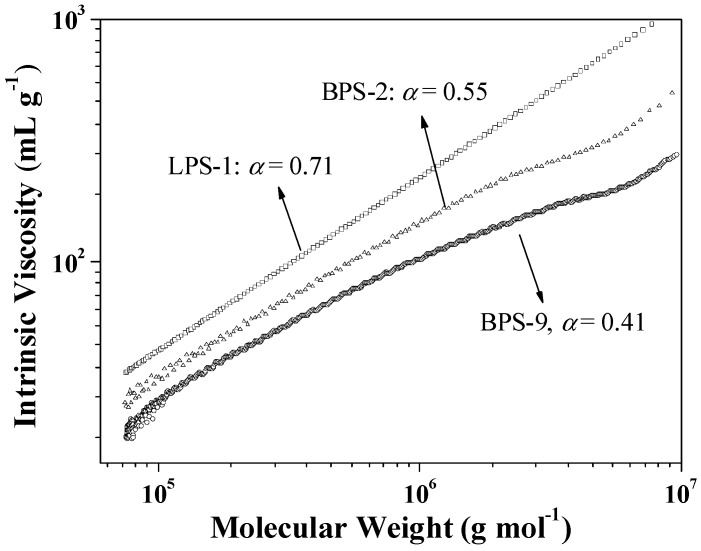
The Mark–Houwink plots of some typical polymers.

The Mark–Houwink exponents of α were determined to be 0.71, 0.55, and 0.41 for LPS-1, BPS-2, and BPS-9, respectively (Figure 4). In addition, LPS-1 and BPS-9 exhibited the highest and the lowest viscosities at any given molecular weight, respectively. These results coincide well with those shown in Figure 3, and furthermore confirm the branching structure of the polymers prepared through radical suspension polymerization in the presence of MHM [5].

With regard to polymer branching structure, we obtained the intrinsic viscosity (IV) and mean-square radius of gyration (*R*_g_) through TD-SEC analysis of the polymers. It is noted that the samples for branching structure information were fractions with relatively narrow polydispersity through polymer fractionation. The Zimm branching factors *g* and *g*′ as the qualitative indicator, as well as the relationship between *g* and *g*′ are given in Equations (1)–(3) [40,41].

*g′ =* IV_Branched_/IV_Linear_ (1)

*g* = <*R*_g_^2^>_Branched_/<*R*_g_^2^>_Linear_ (2)

*g*′ = *g^b^* (3)

**Figure 5:**
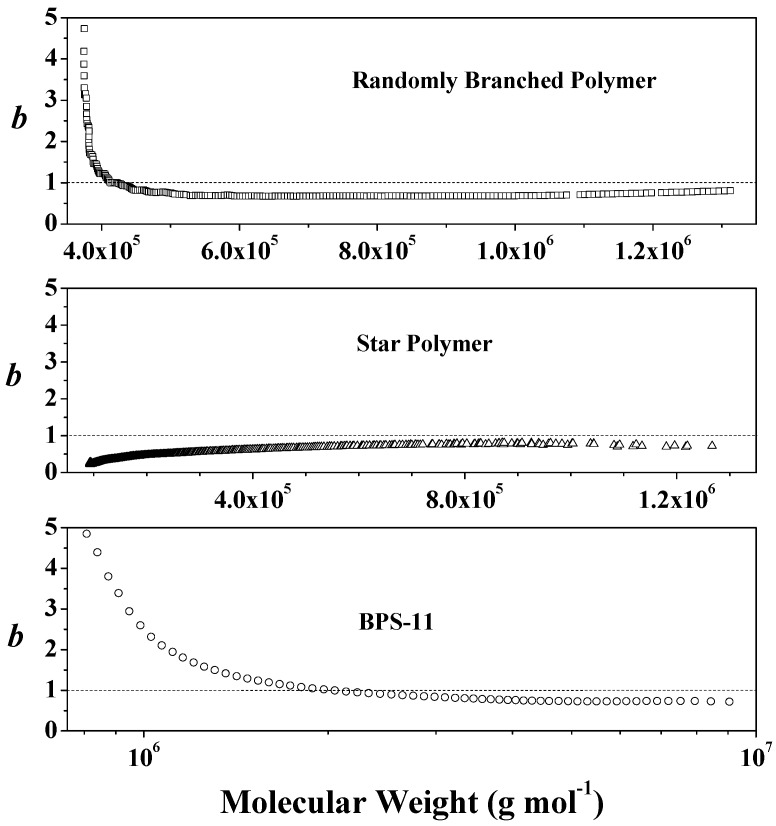
Changes in the exponent *b* with molecular weight for branched polymers.

For regular star polymers, the exponent *b* is less than 1, and it is equal to 0.5 in θ condition and 0.6–0.8 in good solvent [26,40,41]. For randomly branched polymers, the exponent *b* has a starting value larger than 1 and decreases with molecular weight [26,40]. Figure 5 illustrates the changes in the exponent *b* with molecular weight of typical branched polystyrene prepared here (BPS-11, as an example), the reference star, and randomly branched polystyrene in the literature [26], which proves that polymers prepared in the presence of MHM through radical suspension polymerization were of randomly branched structure.

### 3.2. Properties of the Branched Polymers

**Figure 6:**
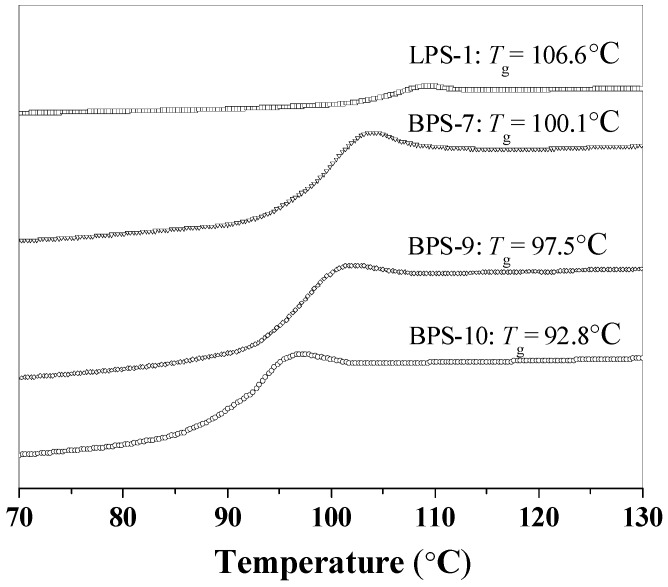
Differential scanning calorimetry (DSC) curves of the linear and branched polystyrenes.

Figure 6 shows the DSC curves of the polymers prepared with and without MHM at different concentrations. Similar to the results in the literature [42], linear polystyrene (LPS-1) showed the highest glass transition temperature (*T*_g_ = 106.6 °C) compared to all three branched polystyrenes (BPS-7, BPS-9, and BPS-10) prepared here, because branched polymers had more chain ends and their polymer segments were more mobile [43]. Additionally, the MHM used in this study is a soft monomer compared to styrene. At a given initiator concentration, polymer prepared at higher MHM concentration showed lower glass transition temperature than that prepared at lower MHM concentration (*T*_g_ = 92.8 °C for BPS-9 vs. *T*_g_ = 100.1 °C for BPS-7). Besides, the higher degree of branching of BPS-9 illustrated some higher soft monomer concentration present in BPS-9 than that of BPS-7. Thus, the higher degree of branching should be the determining factor that accounts for lower glass transition temperature of BPS-9 compared to BPS-7, because polymers with higher degree of branching would have more polymer chain ends at the same molecular weight. At a given MHM concentration, polymer prepared at lower initiator concentration exhibited higher glass transition temperature compared with that obtained at higher initiator concentration (*T*_g_ = 97.5 °C for BPS-9 vs. *T*_g_ = 92.8 °C for BPS-10). The reason for the above is that the primary chain length would be shorter at higher initiator concentration, and therefore the polymer segments would be more mobile, leading to lower glass transition temperature with higher initiator concentration.

**Figure 7:**
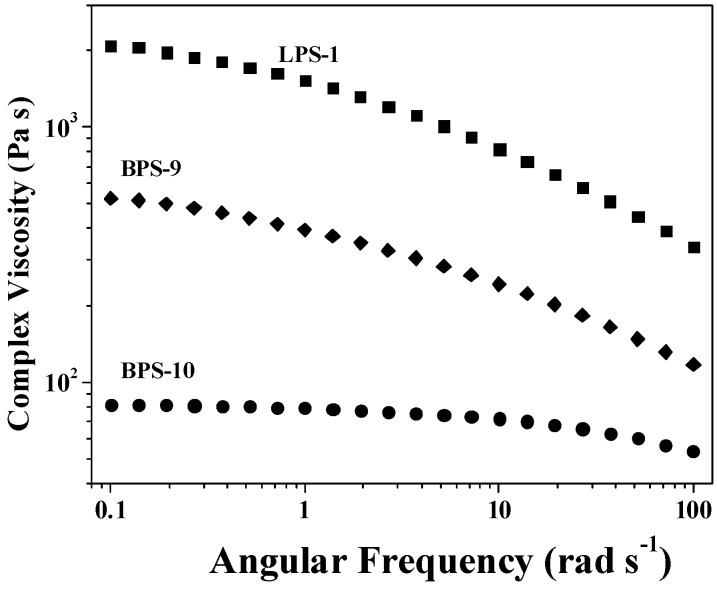
Variation of the complex viscosity with angular frequency of the polymers.

Figure 7 shows a typical variation of the complex viscosity with angular frequency for LPS-1, BPS-9, and BPS-10 samples. Although BPS-9 had much higher molecular weight compared to that of LPS-1 (Table 1), the latter showed much higher complex viscosity at any given angular frequency and evident shear thinning compared to BPS-9, demonstrating that the chain entanglement in the branched polymer was much lower than that in linear polymer. For BPS-9, the decrease in complex viscosity with angular frequency confirmed the existence of chain entanglement and disentanglement with increased angular frequency. However, the complex viscosity of BPS-10 was much lower than those of BPS-9 and LPS-1, but almost no decrease in complex viscosity with angular frequency was observed in the former, implying there was almost no chain entanglement in the branched polymer of low molecular weight. Further investigation also provided evidence for the dependency of chain entanglement on molecular weight in branched polymers (Figures S4 and S5).

## 4. Conclusions

Branched polystyrene beads were successfully prepared directly through solvent-free suspension polymerization using 3-mercapto-hexyl methacrylate (MHM) as the branching monomer and 2,2′-azobisisobutyronitrile (AIBN) as the initiator. Compared with solution polymerization, soluble branched polymer beads were prepared at MHM/AIBN feed ratios less than 1 without any introduction of solvent, because water effectively transfers the polymerization heat. The incorporation of MHM into the polymer was confirmed by FTIR and NMR measurements, and TD-SEC analysis provided evidence of the branching structure of the obtained polymers. Furthermore, polymers prepared at higher MHM concentration exhibited a higher degree of branching. Compared with their linear analogues, lower glass transition temperature was observed in branched polymers because of the introduction of branching. The complex viscosity of branched polymers was much lower than those of linear polymers, suggesting decreased chain entanglement in branched molecules. Furthermore, almost no decrease in complex viscosity with angular frequency was observed in branched polymers of low molecular weight, implying that there was almost no chain entanglement in branched polymers of low molecular weight. Thus, there was also a dependency of chain entanglement on molecular weight in branched polymers.

## Figures and Tables

**Table 1:** Triple Detection Size Exclusion Chromatography (TD-SEC) results of the obtained polymers.

Sample No.	Feed ratio	*M*_n.SEC_^a^ (g·mol^−1^)	*M*_w.MALLS_^b^ (g·mol^−1^)	PDI
LPS-1	St_100_-AIBN_0.5_	50,700	191,400	3.53
LPS-2	St_100_-MHIB_0.5_-AIBN_0.5_	12,900	123,100	6.42
BPS-1	St_100_-MHM_0.25_-AIBN_0.5_	44,300	389,200	5.29
BPS-2	St_100_-MHM_0.5_-AIBN_0.5_	49,500	795,600	6.96
BPS-3	St_100_-MHM_0.75_-AIBN_0.5_	gelation		
BPS-4	St_100_-MHM_1.0_-AIBN_0.75_	gelation		
BPS-5	St_100_-MHM_1.0_-AIBN_1.0_	47,800	723,000	6.04
BPS-6	St_100_-MHM_1.0_-AIBN_1.5_	22,600	289,700	6.19
BPS-7	St_100_-MHM_1.0_-AIBN_2.0_	18,700	256,400	6.50
BPS-8	St_100_-MHM_1.0_-AIBN_2.5_	15,000	96,800	4.29
BPS-9	St_100_-MHM_1.5_-AIBN_2.0_	33,600	1,036,000	7.76
BPS-10	St_100_-MHM_1.5_-AIBN_3.0_	15,700	219,300	6.33
BPS-11	St_100_-MHM_2.0_-AIBN_3.0_	28,100	731,900	7.87
BPS-12 ^c^	St_100_-MHM_2.0_-AIBN_0.5_	32,900	985,400	10.26
BPS-13 ^d^	St_100_-MHM_2.0_-PPDS_0.5_	144,900	2,184,000	4.42

^a^ *M*_n.SEC_ is the number-averaged molecular weight determined by the differential refraction detector size exclusion chromatography. ^b^ *M*_n.MALLS_ is the weight-averaged molecular weight determined by the multi angle laser light scattering detector size exclusion chromatography. ^c^ Polymer prepared through solution polymerization [37]. ^d^ Polymer prepared through emulsion polymerization [39]. BPS: branched polystyrene; LPS: linear polystyrene; PPDS: potassium peroxodisulfate.

## Supplementary Materials

The supplementary materials are available online at www.mdpi.com/2073-4360/9/1/14/s1.
