# Supplementary material for: Preparation and Properties of Branched Polystyrene through Radical Suspension Polymerization
Source: Polymers (Basel). 2017 Jan 6;9(1):14. doi: 10.3390/polym9010014 (PMC6432501; doi:10.3390/polym9010014)
Supplement: Supplementary file 1 [file polymers-09-00014-s001.pdf]

Supplementary Materials: Preparation and Properties of Branched Polystyrene through Radical Suspension Polymerization

Wenyan Huang, Weikai Gu, Hongjun Yang, Xiaoqiang Xue, Bibiao Jiang, Dongliang Zhang, Jianbo Fang, Jianhai Chen, Yang Yang and Jinlong Guo

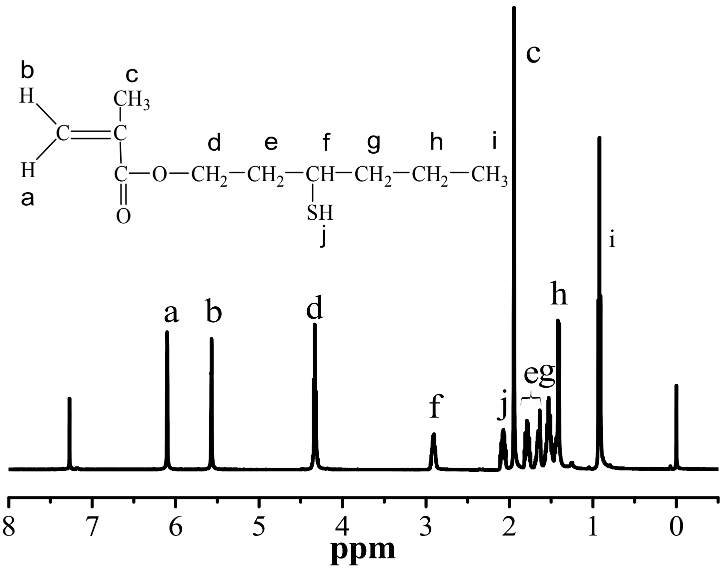

Figure S1. The ^{1}H NMR spectrum of MHM. The spectrum was obtained at 25 °C with $CDCl_3$ as the solvent.

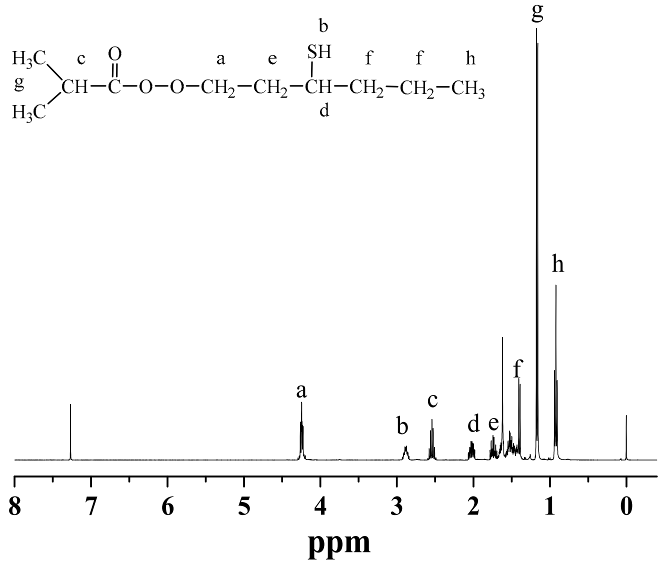

Figure S2. The ^{1}H NMR spectrum of MHIB. The spectrum was obtained at 25 °C with $CDCl_3$ as the solvent.

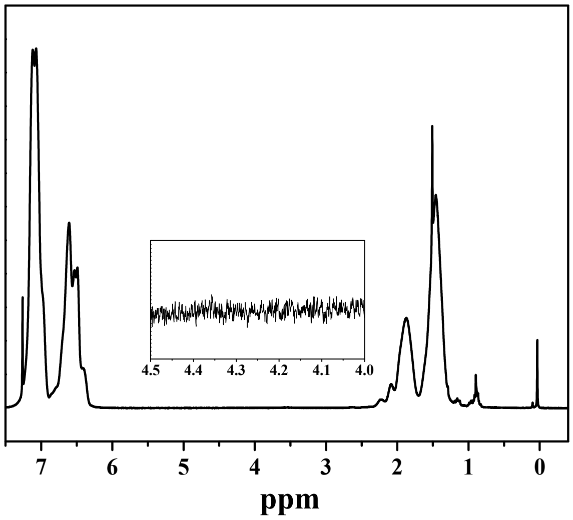

Figure S3. ^{1}H-NMR spectrum of LPS-1 in Table 1, St100-AIBN0.5.

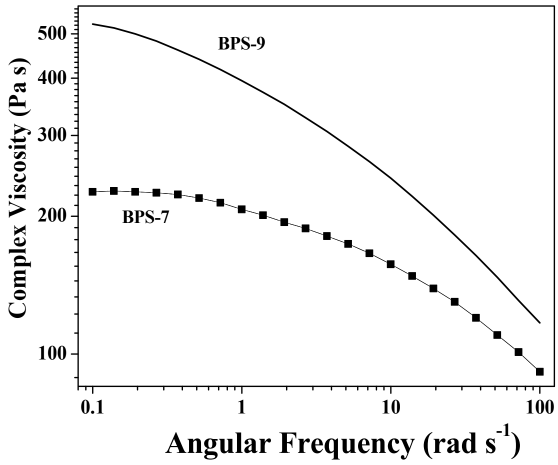

Figure S4. Variation of the complex viscosity with angular frequency of the polymers.

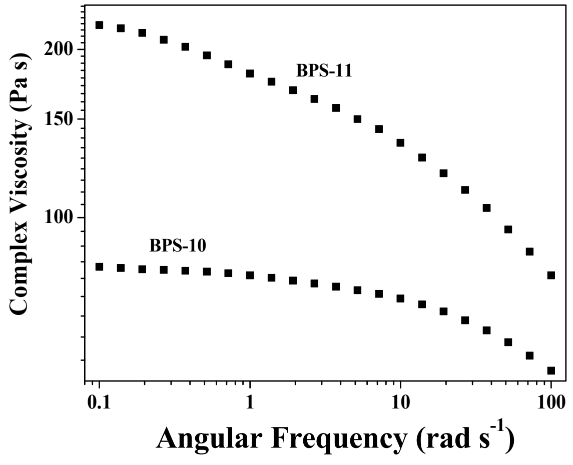

Figure S5. Variation of the complex viscosity with angular frequency of the polymers.
